# Directly Reprogrammed Neurons Express *MAPT* and *APP* Splice Variants Pertinent to Ageing and Neurodegeneration

**DOI:** 10.1007/s12035-020-02258-w

**Published:** 2021-01-07

**Authors:** Mette Habekost, Per Qvist, Mark Denham, Ida E. Holm, Arne Lund Jørgensen

**Affiliations:** 1 Department of Biomedicine, Aarhus University, 8000C Aarhus, Denmark; 2 Danish Research Institute of Translational Neuroscience, Nordic EMBL Partnership for Molecular Medicine, Aarhus University, 8000C Aarhus, Denmark; 3 iPSYCH, Lundbeck Foundation Initiative for Integrative Psychiatric Research, 8000C Aarhus, Denmark; 4 Center for Genomics and Personalized Medicine, 8000C Aarhus, Denmark; 5 Department of Pathology, Randers Hospital, 8930 Randers, Denmark; 6 Department of Clinical Medicine, Aarhus University, 8000C Aarhus, Denmark

**Keywords:** Induced neurons, Splicing, Tau, APP, Alzheimer’s disease, Porcine models

## Abstract

Neurons produced by reprogramming of other cell types are used to study cellular mechanisms of age-related neurodegenerative diseases. To model Alzheimer’s disease and other tauopathies, it is essential that alternative splicing of the *MAPT* transcript in these neurons produces the relevant tau isoforms. Human neurons derived from induced pluripotent stem cells, however, express tau isoform compositions characteristic of foetal neurons rather than of adult neurons unless cultured in vitro for extended time periods. In this study, we characterised the dynamics of the *MAPT* and *APP* alternative splicing during a developmental time-course of porcine and murine cerebral cortices. We found age-dependent and species-specific isoform composition of *MAPT*, including 3R and 4R isoforms in the porcine adult brain similar to that of the adult human brain. We converted adult and embryonic fibroblasts directly into induced neurons and found similar developmental patterns of isoform composition, notably, the 3R and 4R isoforms relevant to the pathogenesis of Alzheimer’s disease. Also, we observed cell-type-specific isoform expression of *APP* transcripts during the conversion. The approach was further used to generate induced neurons from transgenic pigs carrying Alzheimer’s disease-causing mutations. We show that such neurons authentically model the first crucial steps in AD pathogenesis.

**Supplementary Information:**

The online version contains supplementary material available at 10.1007/s12035-020-02258-w.

## Introduction

Neurodegenerative diseases classified as tauopathies have in common intraneuronal accumulation of different isoforms of the microtubules associated protein tau (MAPT) characteristic of each disease. Alzheimer’s disease (AD) is a tauopathy where age-related neurodegeneration results in progressive dementia. The neuropathological findings include characteristic extraneuronal deposits in neuritic plaques of the amyloidogenic Aβ fragment, development of intracellular neurofibrillary tangles (NFTs) of, in particular, tau isoforms with three and four microtubules-binding repeats (3R, 4R), and neuronal death [1–3]. The pathogenic sequence of events is still a matter of dispute, but a long-held hypothesis points to increased production or decreased clearance of the Aβ fragment from the brain as a prime driver of Aβ deposition and subsequent NFT pathology [4, 5].

The vast majority of AD cases have multifactorial aetiology, and more than 50 susceptibility loci with genome-wide significance are associated with the disease [6, 7]. In few cases, the disease behaves in an autosomal dominant fashion and is associated with mutations in the amyloid precursor protein (*APP*) or the presenilin (*PSEN1* or *PSEN2*) genes [8–10].

APP is an integral membrane protein expressed in many cell types. Three main isoforms exist due to alternative splicing and encode proteins of 695, 751 and 770 amino acids (APP695, APP751 and APP770) with APP695 being the typical isoform in neurons [11, 12]. Aberrant processing of APP by the proteolytic enzymes, β- and γ-secretases generates Aβ, and mutations in *APP* and in *PSEN1*/*2* increase the production of Aβ or create more of the hydrophobic 42 amino acids long Aβ peptide (Aβ42) relative to the shorter Aβ40 [13, 14].

Tau belongs to the family of microtubule-associated proteins that also includes MAP2. Six isoforms of tau are produced by alternative splicing of *MAPT*. These isoforms differ by presence or absence of protein domains encoded by exon 2 and exon 3 in the N-terminal part (0N, 1N, 2N), and inclusion (4R) or exclusion (3R) of a microtubule-binding repeat encoded by exon 10 in the C-terminal part of tau [15]. Alternative splicing of *MAPT* is developmentally regulated, and not conserved between species. During human development, only 0N3R tau is expressed, whereas 3R and 4R tau isoforms are expressed at equal levels in the adult brain [16, 17]. Among mammals, only 4R tau is present in the adult rodent brain while both 3R and 4R tau isoforms appear to be expressed in the adult porcine brain [18, 19].

Typically, animal models of AD are generated in mice that have been genetically modified by introducing disease-causing mutations in *APP* or *PSEN1/2*. Such animals are expected to model both the autosomal dominant form and the multifactorial form of AD since the two forms of AD are indistinguishable with respect to clinical and neuropathological phenotypes. However, a serious drawback of these models has been their resistance to develop tau pathology in the form of NFT in the brain. This lack of a key neuronal lesion may be explained by the absence of 3R tau in mouse neurons since the tau isoform composition appears to be critical for filament formation [3, 20].

Human-induced pluripotent stem cells (iPSCs) serve as an attractive cellular alternative as this technology allows patient-specific neurons to be derived from fibroblasts. Indeed, neurons derived from iPSCs that originate from the fibroblasts of patients with AD-causing mutations exhibit aberrant Aβ production [21]. However, it is a challenge to recapitulate tau isoform composition and hence pathology in such cells in vitro. Indeed, several studies have demonstrated the presence of foetal-only (0N3R) tau in wild-type iPSC-derived neurons during in vitro differentiation [22–24], although neurons with frontotemporal dementia (FTDP-17T)-causing *MAPT* mutations mature and induce 4R tau expression faster than control neurons [25]. Accelerated maturation and adult tau splicing have also been achieved in wild-type neurons by enhancing the microenvironment using 3D culturing or *in vivo* transplantation [26, 27]. In this study, we characterise the dynamics of *MAPT* and *APP* alternative splicing during porcine and murine embryonic brain development and demonstrate that directly reprogrammed porcine neurons model age-dependent and cell-type-specific patterns of *MAPT* and *APP* isoform expression allowing in vitro modelling of ageing processes relevant for neurodegenerative disorders.

## Results

### Distinct APP and Tau Isoform Regulation During Murine and Porcine Brain Development

**Fig. 1:**
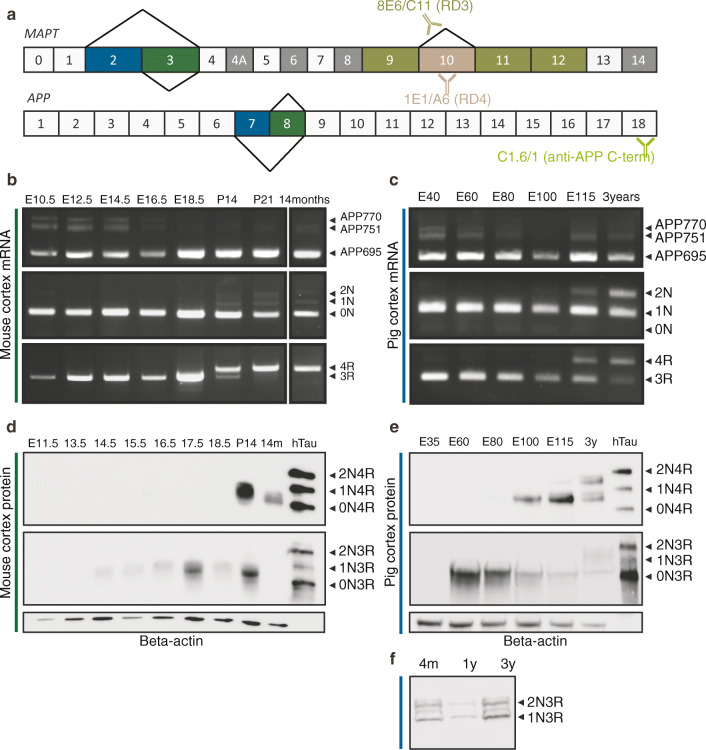
*APP* and *MAPT* isoform expression during murine and porcine brain development. **a** Schematic illustration of *MAPT* and *APP* exon structure. **b**, **c** RT-PCR of *App/APP* and *Mapt/MAPT* isoform expression during embryonic development of cerebral cortex of the mouse (E11.5-E18.5; P14-P21; 14 months old) and the pig (E40–115; 3 years old) using species-specific RT-PCR primers. **d**, **e** Western blotting of protein samples equivalent to **b** and **c** using 3R (RD3; 8E6/C11) and 4R (RD4; 1E1/A6) specific antibodies are recognising tau as illustrated in **a**. Recombinant human tau (hTau) protein marker used as a positive control. Beta-actin used as a loading control. **f** Western blotting of protein samples from adult pig cerebral cortex (4 months; 1 and 3 years old) using 3R (RD3; 8E6/C11) antibody. E, embryonic day/days post-conception; P, postnatal day

Human *APP* has 18 exons, of which exon 7 and 8 are subjects to alternative splicing. The longest isoform is APP770. Exclusion of exon 8 gives rise to APP751, whereas skipping of both exons results in the shortest APP695 variant (Fig. 1a). As for *MAPT*, exclusion of exon 2 and 3 gives rise to 0N tau isoforms, whereas inclusion of exon 2 or exon 2 and 3 makes up the 1N and 2N isoforms, respectively. These three isoforms contain four (4R) or three (3R) microtubule-binding repeat domains depending on exon 10 inclusion or exclusion, respectively, and give rise to six main tau isoforms: 0N3R, 1N3R, 2N3R and 0N4R, 1N4R, 2N4R (Fig. 1a). Corresponding exon organisation for mouse and porcine *Mapt/MAPT* and *App/APP* were found using the Ensembl genome browser and are defined in Supplementary Table 1–3.

**Fig. 2:**
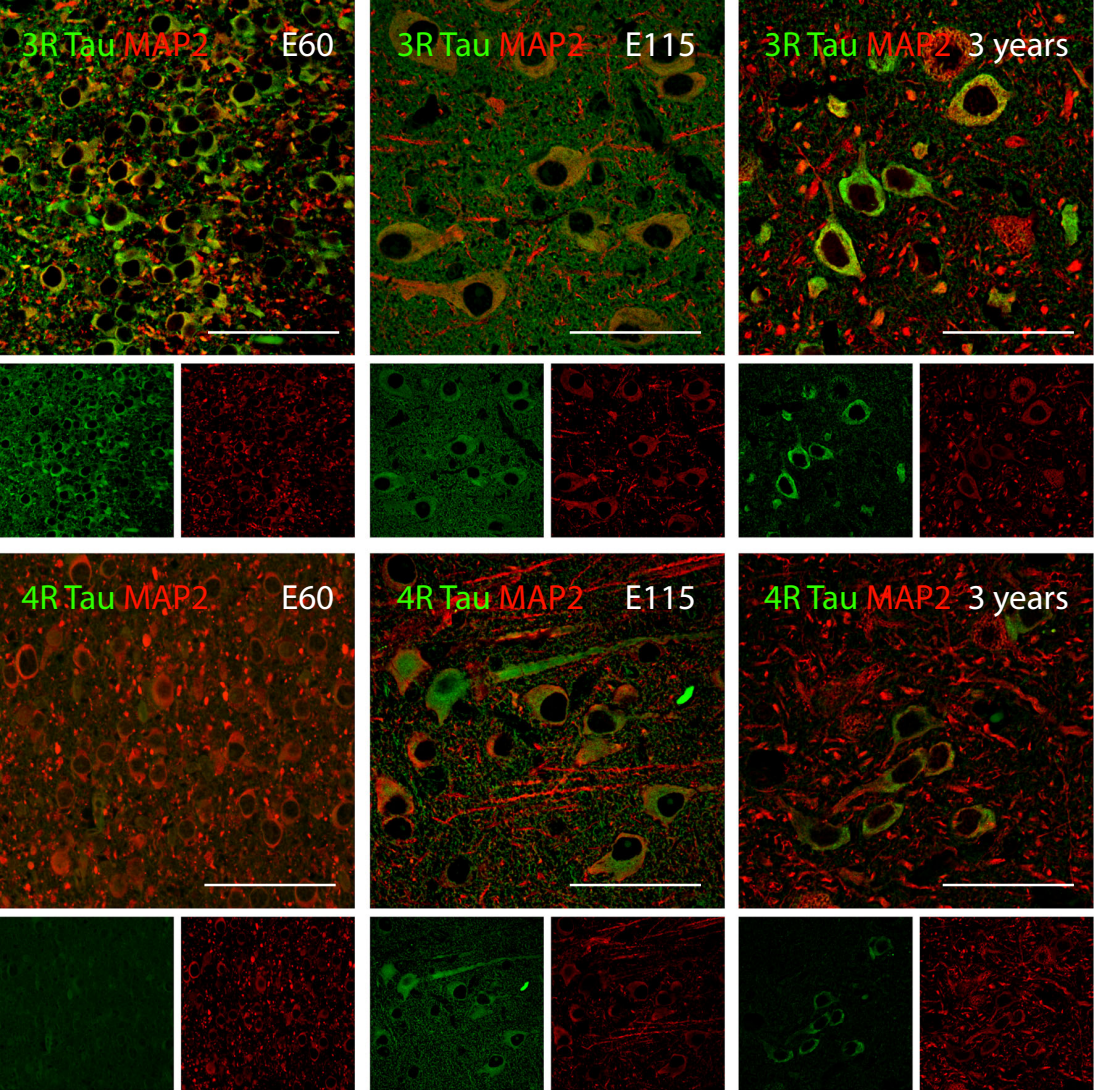
MAPT cell-type specific isoform expression during porcine brain development. Immunohistochemical analysis of MAPT isoform expression in the cerebral cortex of the pig at different ages (E60; E115; 3 years old) using 3R (RD3; 8E6/C11) and 4R (RD4; 1E1/A6) specific antibodies (green). Neurons identified using MAP2 specific antibody (red). E60 showed neuronal staining of 3R tau. The same was true for E115 and 3 years which, in addition, stained positive for 4R tau. Scale bars, 50 μm. E, embryonic day

To determine isoform expression changes during embryonic development of the mouse and the pig brains, we used specimens representing foetal and adult cerebral cortex. We extracted RNA and protein from mice ranging in ages from embryonic day 10.5 (E10.5) to E18.5, postnatal day 14 (P14), P21, and from a 14-month-old adult mouse (Fig. 1b). Likewise, we extracted RNA and protein from E35 to E115 and from one 3-year-old adult pig (Fig. 1c). Species-specific RT-PCR primers were designed to detect *APP/App* and *MAPT/Mapt* isoforms. During mouse cortical development from E10.5 to E18.5, we exclusively detected 0N3R tau isoform (Fig. 1b). At P14, a clear shift to 4R tau was observed while, at the same time, 3R tau had decreased to a low level. At this time point, faint bands emerged representing 1N and 2N, but 0N remained dominant as indicated by the corresponding strong band, and from P21, the 0N4R tau isoform was the predominant transcript variant detected (Fig. 1b). During porcine development from E40 to E100, we detected 1N3R and found that the porcine brain expressed 2N and 1N as well as 3R and 4R tau isoforms from E115 into adulthood (Fig. 1c). Immunoblotting using 3R- and 4R-specific antibodies (RD3 and RD4, respectively; Fig. 1a) confirmed the isoform switch from 0N3R to 0N4R in the mouse protein samples and the gradual switch from 1N3R to the four isoforms 1N3R, 1N4R, 2N3R, and, 2N4R in the pig protein samples (Fig. 1d–f). Immunohistochemical staining of cortical sections showed that tau isoforms were expressed in neurons (MAP2+ cells) (Fig. 2, Supplemental Figure 1). With respect to *APP/App*, the isoforms APP770 and APP751 were detected at low and decreasing levels while APP695 was the predominant isoform in both species throughout the time-course (Fig. 1b–c). As a reference, we detected six *MAPT* and three *APP* isoforms in the adult human cortex (Supplemental Figure 2A).

### Dynamic Isoform Expression Pattern of APP and Tau During Conversion of Porcine-Induced Neurons

**Fig. 3:**
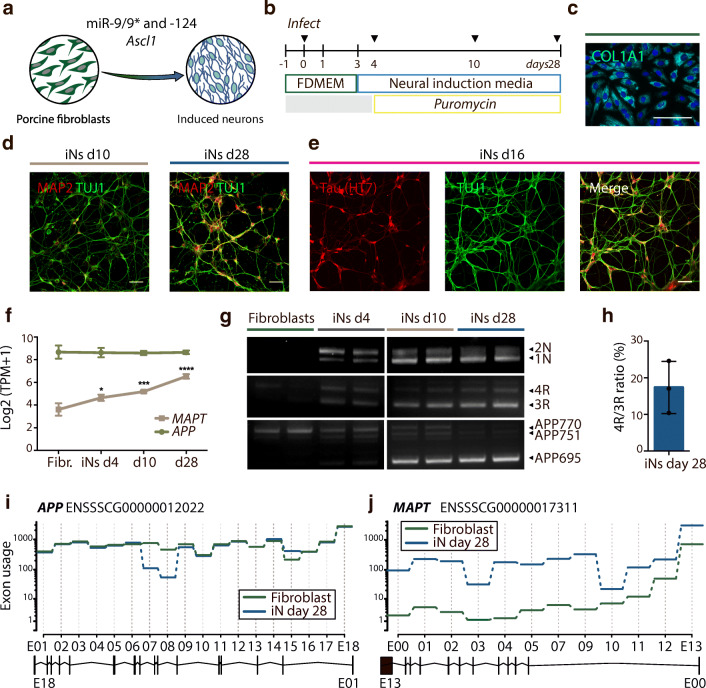
Direct conversion of porcine fibroblasts into induced neurons. **a**, **b** Schematic illustration of conversion strategy. Wild-type porcine fibroblasts converted into induced neurons by overexpression of *mir-9* and *mir-124* (mir-9/9*-124) and *Ascl1* cultured in neural induction media with puromycin selection up to day 28 after transduction. Arrows in **b** indicate time points for RNA extraction. **c** Immunostaining of fibroblasts for fibroblast marker COL1A1 (cyan). **d** Immunostaining of iNs for pan-neuronal TUJ1 (green) and mature marker MAP2 (red) after 10 and 28 days of conversion (d10, d28). **e** Immunostaining of iNs for TUJ1 (green) and tau (red, all isoforms) after 16 days of conversion (d16). All immunostainings counterstained with DAPI (blue). Scale bars, 50 μm. **f** *APP* and *MAPT* expression level profiling (Log2(TPM+1)) during the conversion of fibroblasts into neurons at time points indicated in **b**. Results presented as mean ± s.d., *n* = 3 for each time point. Two-way ANOVA followed by Dunnett’s multiple comparisons test. **g** RT-PCR of *APP* and *MAPT* isoforms during neuronal conversion using species-specific RT-PCR primers. *n* = 2 for each time point. **h** Quantification of junction read counts of 3R and 4R tau isoforms presented as 4R/3R ratio of iNs 28 days post-transduction. Result presented as mean ± s.d., *n* = 3. (I, J) DEXSeq analysis quantifying differential *APP* and *MAPT* exon usage between fibroblasts (green) and iNs 28 days after conversion (blue). Dotted lines in dark grey indicate differential exon usage. Ensembl IDs and exon structures for *APP* and *MAPT* are shown. E, exon

Porcine wild-type fibroblasts from a 4-month-old animal (PAF4M) were converted into induced neurons (iNs) by overexpression of mir-9/9*-124 and *Ascl1* supported by small molecules as previously described [28](Fig. 3a–b). The starting fibroblast population stained positive for COL1A1 (Fig. 3c), a marker shown to be present in pig fibroblasts and absent in iNs [28]. Ten days post-transduction, the cells were immuno-positive for the pan-neuronal marker β-tubulin III (TUJ1) and mature neuronal marker MAP2 and with increased staining over time (Fig. 3d). At day 16 post-transduction, the cells became immune-positive for protein tau, which appeared located in soma and neurites at this time point (Fig. 3e).

To examine *APP* and *MAPT* isoform expression changes during neuronal conversion, we first assessed gene expression levels of *APP* and *MAPT* in fibroblasts and iNs at day 4, 10 and 28 using RNA sequencing (Fig. 3b, f). We found *APP* to be expressed at equal levels across samples, whereas the expression level of *MAPT* significantly increased as the cells converted into iNs (Fig. 3f). We next characterised the isoform composition of *MAPT* and *APP* using RT-PCR primers as in Fig. 1. This revealed dynamic alternative splicing events (Fig. 3g). While the gene expression level of *APP* did not change over time (Fig. 3f), the dominant transcript noticeably changed from APP770 in fibroblasts to APP695 in iNs (Fig. 3g). The shift in isoform expression occurred between day 4 and 10, at which time points both APP770 and APP751 were still detectable (Fig. 3g). *MAPT* expression was barely detectable in fibroblasts but increased to day 4, where 2N, 1N, 3R and 4R tau isoforms were clearly detected. This isoform composition did not change from day 4 to day 28, although the relative amount of 1N and 3R isoforms appeared to increase over time (Fig. 3g). DEXSeq analysis quantifying exon usage revealed significant differences in usage of *APP* exon 7 and 8 while the overall exon usage remained the same between fibroblasts and d28 iNs (Fig. 3i), which is in accord with the RT-PCR results, shown in Fig. 3g. Usage of all *MAPT* exons was significantly increased in iNs compared to fibroblasts (Fig. 3j). The analysis showed that exon 2, 3 and 10 were expressed in the iNs, confirming the RT-PCR results (Fig. 3g). Immunostaining of iNs after 28 days of conversion with 3R- and 4R-specific antibodies confirmed the isoform composition (Supplementary Figure 3). Exon junction counts revealed a 4R/3R tau ratio of 17.34 ± 7.11% (s.d.) (Fig. 3h). This encouraged us to look into the tau isoform composition of human-derived iNs. We utilised publicly available data on global gene expression of human iNs converted using *Ngn2* and *Ascl1* (N2AA) or shREST, mir-9/9*-124, *Brn2* and *Ascl1* (REST_sh-PBmPA) protocols [29, 30]. Visual inspection of the data with Integrative Genomics Viewer (IGV) revealed that all six *MAPT* isoforms were expressed in both datasets (Supplemental Figure 2B-E). Counting junction reads revealed a 4R/3R tau ratio of 51.02 ± 22.13% (s.d., *n* = 2) for N2AA and 108.3 ± 58.93% (s.d., *n* = 2) for REST_sh-PBmPA converted iNs.

### Differential Tau Isoform Composition in Induced Neurons from Embryonic and Adult Fibroblasts

**Fig. 4:**
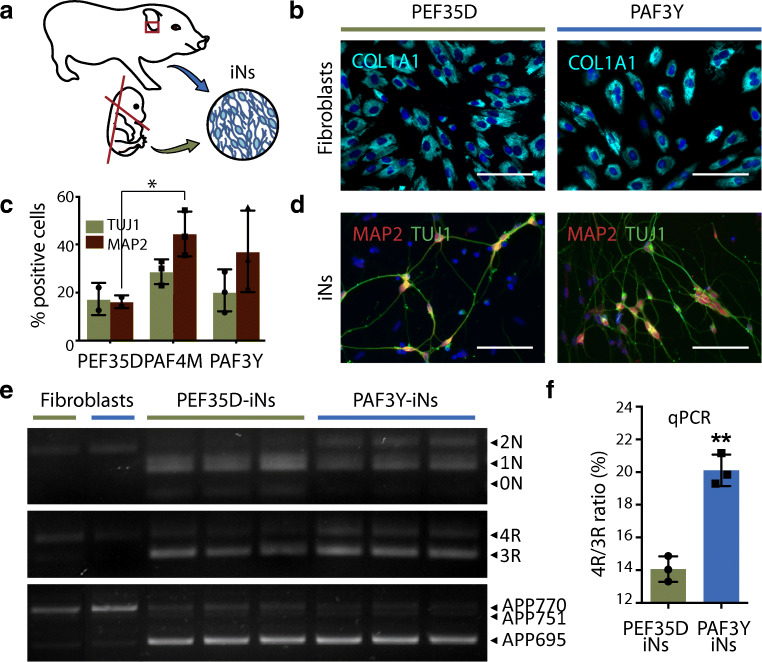
Direct conversion of porcine embryonic fibroblasts into induced neurons. **a** Schematic illustration of direct conversion of porcine embryonic (E35) and adult (3 years) fibroblasts. **b** Immunostaining of embryonic (PEF35D, green) and adult (PAF3Y, blue) fibroblasts for fibroblast marker COL1A1 (cyan). **c** Quantification of the number of TUJ1 (green) and MAP2 (red) positive PEF35D (*n* = 2), PAF4M (porcine adult fibroblasts 4 months, *n* = 3) and PAF3Y (*n* = 3) fibroblast-derived induced neurons 21 days after conversion. Results presented as percentage (%), mean ± s.d.. Two-way ANOVA followed by Sidak’s multiple comparisons test. **d** Immunostaining of PEF35D- and PAF3Y-derived iNs after 21 days of conversion for TUJ1 (green) and MAP2 (red). All immunostainings counterstained with DAPI (blue). Scale bars, 50 μm. **e** RT-PCR of *APP* and *MAPT* isoform expression in PEF35D, PAF3Y and derived iNs using species-specific RT-PCR primers. *n* = 1 for fibroblasts, *n* = 3 for iNs. **f** RT quantitative PCR of 3R and 4R tau isoforms using exon junction-specific primers. Results presented as mean ± s.d., *n* = 3 for each sample. Unpaired *t* test

We next converted porcine fibroblasts derived from E35 embryos (PEF35D) and from 3-year-old adult animals (PAF3Y) into iNs (Fig. 4a) and evaluated the iNs at day 21 post-transduction. All fibroblasts in the starting population stained positive for COL1A1 (Fig. 4b). Conversion rates were assessed by staining for the presence of pan-neuronal markers β-tubulin III (TUJ1) and MAP2 (Fig. 4c–d). The protocol converted PEF35D, PAF4M and PAF3Y into iNs with similar rates, although significant lower MAP2 percentages were observed for PEF35D compared to adult fibroblast conversions (Fig. 4c). We then evaluated the isoform composition of *APP* and *MAPT* in fibroblasts and iNs from PEF35D and PAF3Y (Fig. 4e). As in previous results, we found that *MAPT* expression level increased during conversion from fibroblasts to iNs. In PEF35D-iNs, 1N3R tau isoform appeared to be the dominant *MAPT* transcript (Fig. 4e). By contrast, iNs derived from PAF3Y fibroblasts expressed similar tau isoforms as did PAF4M-iNs, as we found expression of 2N, 1N, 3R and 4R tau exons in these cells (compare Fig. 3g and Fig. 4e). Because we observed a tendency of adult fibroblasts to convert into iNs at higher rates than embryonic fibroblasts, we quantified and compared the expression level of 4R tau as a ratio between 4R and 3R. This revealed a significantly lower 4R/3R ratio in PEF35D-iNs compared to PAF3Y-iNs (Fig. 4f). PAF3Y-iNs exhibited a 4R/3R ratio of 20.1 ± 0.96% (s.d.) similar to the tau ratio determined by junction counts of PAF4M-iNs (compare Fig. 3h and Fig. 4f). In addition, cell-type-specific but not age-dependent differences in *APP* isoform composition were detected (Fig. 4e).

### Generation of Induced Neurons from Porcine Models of Alzheimer’s Disease

**Fig. 5:**
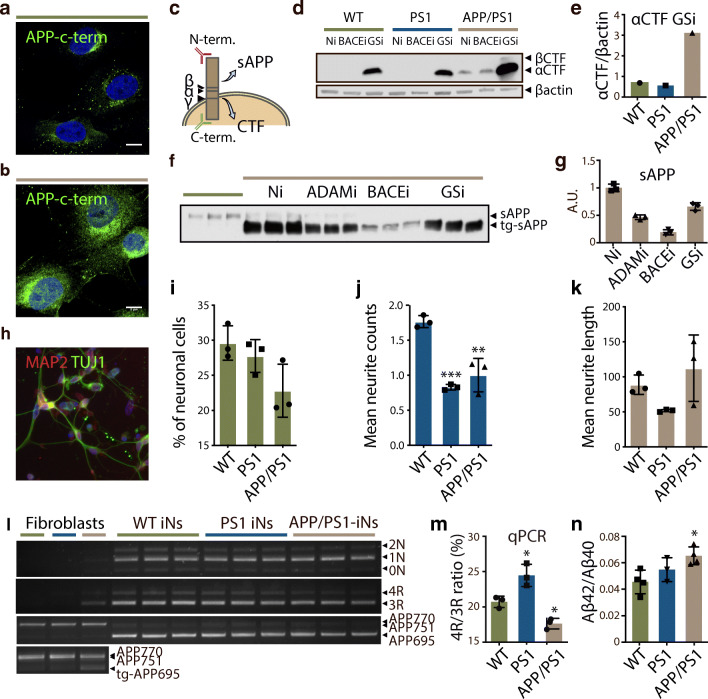
Direct conversion of porcine fibroblast from transgenic models of Alzheimer’s disease into induced neurons. **a**, **b** Immunostaining of fibroblasts from wild-type (WT) and double-transgenic APP/PS1 minipigs for APP (C1/6.1 antibody, green) recognising C-terminal part of APP as illustrated in **c**. Counterstained with DAPI (blue). Scale bar, 8 μm. **c** Schematic illustration showing transmembrane localisation of APP, as well as cleavage sites of α-, β- and γ-secretases releasing sAPP and C-terminal fragments. Antibodies recognising N- (clone 22C11) and C- (clone C1/6.1) terminal parts of APP are shown in red and green, respectively. **d** Western blot of APP processing in fibroblast lysates from WT, single- (PS1) and double- (APP/PS1) transgenic minipigs with or without (Ni) γ- (GSi; L685,458; 1 μM) or β- (BACEi; β-secretase IV; 10 μM) secretase inhibition using anti-APP C1/6.1 antibody. β-actin used as a loading control. **e** Quantification of αCTF bands in (D). *n* = 1 for each sample. **f** Western blot of sAPP-conditioned media from triplicates of WT and double-transgenic minipig with or without α- (ADAMi; TAPI-1; 10 μM), β- (BACEi; β-secretase IV; 10 μM) and γ- (GSi; L685,458; 1 μM) secretase inhibition using anti-APP antibody 22C11. **g** Quantification of transgenic (tg) sAPP in **f**. Results presented as mean ± s.d., *n* = 3 for each sample. **h** Representative immunostaining of transgenic pig-derived iNs for pan-neuronal TUJ1 (green) and mature marker MAP2 (red). Counterstained with DAPI (blue). **i**, **j**, **k** Quantification of neuronal cells (TAU+/DAPI), mean neurite counts and mean neurite length. Results presented as mean ± s.d., *n* = 3 for each sample. One-way ANOVA followed by Dunnett’s multiple comparisons test. **l** RT-PCR of *APP* and *MAPT* isoform expression in WT-, PS1- and APP/PS1-derived iNs using species-specific RT-PCR primers. *n* = 1 for fibroblasts, *n* = 3 for iNs. **m** RT quantitative PCR of 3R and 4R tau isoforms using exon junction-specific primers. Results presented as mean ± s.d., *n* = 3 for each sample. One-way ANOVA followed by Dunnett’s multiple comparisons test. **n** ELISA measurements of Aβ-40 and -42 in media of WT, PS1 and APP/PS1 fibroblasts and iNs after 28 days of conversion. Results presented as mean ± s.d., *n* = 4 (WT; APP/PS1) or 3 (PS1) for each sample. One-way ANOVA followed by Dunnett’s multiple comparisons test

In order to demonstrate the applicability of iNs as a relevant disease model in neurodegenerative disorders, we generated and characterised iNs from fibroblasts from transgenic porcine models carrying AD-causing mutations in *APP* (Swedish mutation; *APP*sw) and/or *PSEN1* (*PSEN1M146I*) (skin biopsies from 12 to 20 weeks old pigs) [31, 32]. Immunofluorescence of fibroblasts from wild-type (WT fibroblasts) and double-transgenic APP/PS1 pigs (APP/PS1 fibroblasts) revealed distinct APP expression levels with the highest level in APP/PS1 fibroblasts due to overexpression of the human transgene *APP*695sw in this model (compare Fig. 5a and b). The punctate staining of APP demonstrated a perinuclear and vesicular localisation indicative of correct synthesis and trafficking of the protein. Electrophoresis and immunoblotting using APP C-terminal antibody (Fig. 5c) did not detect any APP or APP derived fragments in WT or PS1 fibroblasts, indicating low levels of endogenous APP expression (Fig. 5d; lane WT-Ni and PS1-Ni). After treatment with γ-secretase inhibitor, αC-terminal fragment (αCTF), but not βCTF became detectable, demonstrating that very little endogenous APP was processed by β-secretase (Fig. 5d; lane WT-GSi and PS1-GSi; Fig. 5e). Overexpression of the *APPsw* transgene was evident in double-transgenic APP/PS1 fibroblasts as the accumulation of αCTFs were detected in non-inhibited cells (Fig. 5d; lane APP/PS1-Ni). Upon γ-secretase inhibition, a band representing βCTF was observed (Fig. 5d; lane APP/PS1-GSi). Accordingly, the band of soluble APP (sAPP; consists of sAPPα and sAPPβ migrating as one band) decreased after treatment with both α- and β-secretase inhibitors (Fig. 5c, f, g). The molecular weight difference between the tg-sAPP band of the transgenic pig and the sAPP band of the WT pig represents the amino acid differences between transgenic APP695 and endogenous APP770.

Porcine iNs derived from PS1 and APP/PS1 fibroblasts displayed similar neuronal marker expression and conversion rates as those derived from WT fibroblasts (Fig. 5h–i). However, these iNs exhibited significantly fewer neurites per neuronal cell than WT-derived iNs (Fig. 5j). Neurite lengths did not differ (Fig. 5k).

We next assessed isoform composition of *APP* and *MAPT* in fibroblasts and iNs from WT, PS1 and APP/PS1 fibroblasts. Based on RT-PCR, we detected the expression of 2N, 1N, 3R and 4R tau in all iNs, but also a lower migrating band representing 0N tau was visible (Fig. 5l). We quantified and compared the 4R/3R tau ratio and found significantly higher 4R/3R ratio in PS1-iNs and reduced 4R/3R ratio in APP/PS1-iNs compared to WT-iNs (Fig. 5m). Using pig-specific APP primers, we confirmed that endogenous *APP* in all iN-lines changed from *APP*770 in fibroblasts to *APP*695 in iNs (Fig. 5l). The transgenic APP contribution was found only in APP/PS1 fibroblasts using primers detecting both human and porcine APP (Fig. 5l).

We measured the levels of extracellular Aβ40 and Aβ42 during iN conversion of WT-, PS1- and APP/PS1-derived fibroblasts. The Aβ levels were lower in WT and PS1 fibroblast than in iNs (data not shown). The Aβ42/40 ratio was increased in iNs derived from PS1 and APP/PS1 fibroblasts compared to those of WT pigs (Fig. 5n).

## Discussion

We identified 0N3R and 1N3R as the two main foetal tau isoforms present in the developing brains of mice and pigs, respectively. During human development, 0N3R tau is expressed [15] indicating that the presence of only 3R tau isoforms is conserved during brain development of the three species. This is consistent with the need for neuronal plasticity during development as isoforms with 4 repeats (4R) bind and support microtubules more potently than 3R isoform. The N-terminal parts of the isoform do not function in microtubule assembly [16, 33].

Differential splicing of tau between species was most evident in the adult brains. In the mouse adult brain, one tau isoform, 0N4R, was predominant. By contrast, four tau isoforms, 1N3R, 2N3R, 1N4R and 2N3R, were found in the adult porcine brain, and this composition with 3R and 4R isoforms is more similar to the composition of six isoforms found in the human adult brain where 3R and 4R are expressed at similar levels (Goedert and Jakes, 1990). The time point at which longer N-terminal isoforms started to be expressed was similar to the time point at which 4R isoforms could be detected for both species, suggesting that splicing of these domains is co-regulated.

The iPSC technology allows patient-specific neurons to be derived from somatic cells such as fibroblasts. The process of reprogramming involves rejuvenation as the resultant iPSCs resemble embryonic stem cells in regard to telomere length, mitochondrial metabolism and oxidative damage, which may not be useful for studying age-related pathologies. In addition, neurons derived from in vitro differentiation of iPSC or ESCs display developmental gene splicing as previously demonstrated for *NCAM1* and *MAPT* [22, 23, 34]. In contrast, direct conversion of fibroblasts into induced neurons (iNs) circumvents the pluripotent intermediate state, and it has been demonstrated that iNs retain the transcriptomic and epigenetic age of the starting cells [29, 35]. No authentic porcine embryonic stem cells or iPSCs are available [36, 37], but we recently established a protocol for directly converting porcine fibroblasts into iNs [28]. To our surprise, we found that iNs from adult porcine fibroblasts expressed the same four tau isoforms as do neurons of the adult pig brain. In publicly available RNAseq data, we found that also human fibroblast-derived iNs expressed the same tau isoforms as the adult human brain. To our knowledge, this observation regarding iNs has never been reported before, and we hypothesised that tau isoform composition could be used as a marker of cellular age [25]. To this end, we investigated tau isoforms in porcine embryonic fibroblasts converted into iNs and found that 1N3R, the porcine foetal isoform of tau, was the dominant transcript in these cells. We detected only low levels of 4R tau compared to levels observed in adult porcine-derived iNs.

The human and porcine iNs were converted using different transcription factor combinations, suggesting that the induction of 4R tau isoforms is not related to the choice of direct conversion strategy [25]. These experiments were all based on bulk RNA extractions, and isoform expression analysis remains to be performed at a single-cell level. The 3R and 4R tau antibodies used in this study were both mouse immunoglobulins and, therefore, could not be used together to answer this question.

Tau participates in axonal transport of neurons, and it was recently shown that imbalance in the normal 3R/4R ratio could impair transportation of APP [38], which highlights the importance of correct tau isoform expression to model certain aspects of neurodegeneration. We found cell-type-specific isoform expression of *APP* during conversion from fibroblasts to neurons, i.e. switch of *APP*770 to the neuronal isoform *APP*695 between day 4 and 10 in the conversion process without changing the general expression level of *APP*. Similarly, endogenous APP mRNA in our single- and double-transgenic animals to iNs was spliced normally, and quantification of Aβ detected an increase in the Aβ42/40 ratio in transgenic pigs consistent with the effects of *PSEN1M146I* and *APPsw* transgenes. This means that the first crucial steps in AD pathogenesis are faithfully modelled in these iNs.

The finding that the tau isoform composition in porcine and human-induced neurons resembles that of the adult brain of each species has implications for the choice of the cellular system to use for in vitro modelling and drug screening purposes of age-related neurodegenerative disorders such as Alzheimer’s disease.

## Materials and Methods

### Tissue

Porcine foetuses were obtained from artificially inseminated landrace sows (*Sus scrofa*). Pregnant sows were anaesthetised by inhalation of 35–70% CO_2_ for 1 min and sacrificed by exsanguination. The uteri were recovered and the foetuses from 35/40, 60, 80, 100 and 115 days post-conception were quickly removed, dissected and flash-frozen in liquid nitrogen. Tissue for immunohistochemical analysis was immersed in neutral-buffered 10% formalin (Sigma-Aldrich).

Murine foetuses were obtained from timed mating of mice acquired from Taconic M&B, Ry, Denmark. Females were examined the following morning, and the stage of development was designated embryonic day 0.5 (E0.5). Pregnant mice were killed by cervical dislocation. Foetuses were dissected from the age of embryonic day 10.5, 11.5, 12.5, 13.5, 14.5, 15.5, 16.5, 17.5 and 18.5 (day of birth), and postnatal day 14 (P14) and P21. The anterior part of the embryo was collected from E10.5 to E16.5, and the brains were dissected from E17.5, E18.5, P14, P21 and from a 14-months-old adult mouse. Samples were immediately flash-frozen in liquid nitrogen. Tissue for immunohistochemical analysis was immersed in neutral-buffered 10% formalin (Sigma-Aldrich).

### Cell Culture

Porcine fibroblasts (WT♂#3430 19 weeks old; WT♂#317245 3 years; PS1♂#4902 12 weeks; APP/PS1♀#6036 20 weeks old) were obtained from ear biopsies from Göttingen minipigs and cultured in Dulbecco’s Modified Eagle’s Medium (DMEM; Sigma-Aldrich), 1% FBS (Life Technologies), 1% P/S (P/S, 10,000 units/mL and 10,000 μg/mL; Life Technologies), 1% Gln (2.92 g/100 mL) and 6.667 ng/mL fibroblast growth factor-basic (bFGF; Life Technologies) in 5% CO_2_ in a humidified chamber at 37 °C. The outgrowing fibroblasts were washed with phosphate buffered saline (PBS; Life Technologies) and harvested by trypsinisation for 2–3 min at 37 °C (0.05% trypsin EDTA; Life Technologies) and cultured for one additional passage before long-term storage at − 135 °C. Established fibroblasts were cultured in fibroblasts media and maintained in 5% CO_2_ in a humidified chamber at 37 °C (unless otherwise stated) and split in a 1:3 ratio every 2–3 days using 0.05% trypsin EDTA (Life Technologies).

Porcine embryonic fibroblasts were obtained from Göttingen minipig embryos at day 35 of gestation. The heads of the foetuses were removed together with the remaining internal organs, and the bodies were fragmented and incubated with 0.05% trypsin EDTA (Life Technologies) containing DNAse (1:50; Roche) in 37 °C for 5–10 min and disaggregated by pipetting using a 21G needle (Terumo). Embryonic fibroblasts were maintained as described for porcine adult fibroblasts. All fibroblasts used in this study tested negative for mycoplasma (MYCOPLASMACHECK, Eurofins Genomics). Göttingen minipigs are housed at Research Center Foulum, Department of Animal Science, Aarhus University.

### Lentivirus Production

For direct fibroblast-to-neuron conversion, we used commercially available plasmids pTight-9-124, Tet-O-Ascl1 and FUW-M2rtTA (Addgene plasmid #31874, #27150 and #20342). Lentiviral particles were produced using 2nd generation lentiviral packaging vectors (psPAX2 and pMD2.G, Addgene plasmid #12260 and #12259). In brief, HEK293T/17 (ATCC, cat. no. CRL-11268) cells were transfected with the plasmid of interest together with packaging and envelope plasmids using Lipofectamine™ 3000 Transfection Reagent (Life Technologies). The supernatant containing the virus particles was collected 24 and 48 h post-transfection, filtered (0.45 μm filter, Frisenette), concentrated by ultracentrifugation 22500 g for 1 h and 30 min at 4 °C and resuspended in PBS and snap-frozen and stored at − 80 °C. Lentiviral titration was performed by quantitative PCR using 7500 Fast real-time PCR system (Applied Biosystems) and primers against the lentiviral backbone (LV2) and albumin (*ALB*) as an internal control for normalisation. Titre was calculated by comparing integrated viral DNA content against a vector reference, pWPXL (pWPXL was a gift from Didier Trono) with known titre determined by green fluorescent protein (GFP) expression in transduced cells. All viruses had titre above 1.0 × 10^8^.

### Direct Reprogramming

The direct conversion was performed as previously described [28]. In brief, porcine fibroblasts were plated onto poly-L-ornithine hydrobromide (0.1 mg/mL; Sigma-Aldrich)-, laminin L2020 (10 μg/mL; Sigma-Aldrich)- and fibronectin (10 μg/mL; Sigma-Aldrich)-coated cell culture-treated plastic plates (Fisher Scientific) or acid-treated coverslips (VWR). Fibroblasts were transduced with concentrated lentivirus at the multiplicity of infection 20 of individual vectors. After 16 h, the media was changed and supplemented with doxycycline (DOX; 2 μg/mL; Sigma-Aldrich). On day 3 post-transduction, the media was replaced by neuronal conversion media (Neurobasal media (Life Technologies) and DMEM/F12 (Life Technologies) mixed at a 1:1 ratio containing 1% glucose (30%), 1% P/S (10,000 units/mL and 10,000 μg/mL; Life Technologies), 1% Glutamax (Life Technologies), 1% ITS-A (Life Technologies), 1% B27 (Life Technologies) and 1% N2 (Life Technologies)) supplemented with the small molecules CHIR99021 (2 μM; Stemgent), SB-431542 (10 μM; Tocris), LDN-193189 (0.5 μM; Stemgent), A-83 (0.5 μM; Tocris), Forskolin (5 μM; Sigma-Aldrich) and growth factors LM-22A4 (2 μM; Tocris), GDNF (2 ng/mL; R&D Systems), NT3 (10 ng/mL; Peprotech) and db-cAMP (0.5 mM; Sigma-Aldrich), as well as the epigenetics regulator valproic acid (VPA; 1 mM; Sigma-Aldrich) and antibiotics (puromycin; 1 μg/mL; Gibco). Fibroblasts derived from transgenic animals were puromycin-resistant [31] and all conversions comparing WT to transgenic fibroblast were therefore carried out without puromycin selection but with the addition of AraC (2 μM; Sigma-Aldrich) to inhibit proliferation of non-converting fibroblasts. Half media changes were performed every 2–3 days until analysis.

### Immunocytochemistry

Cells plated on coverslips (VWR) were washed twice with PBS (Life Technologies) and fixated in 4% paraformaldehyde (PFA; Santa Cruz Biotechnologies, Inc.) for 15 min at 4 °C, then washed with PBS (Life Technologies) and distilled water (Life Technologies) and left to dry for 10 min. The cells were then permeabilised for 10 min at RT in PBS (Life Technologies) containing 0.25% Triton X (Sigma-Aldrich) (PBT) and blocked for 1 h at RT in PBT supplemented with 5% donkey serum (Almeco). The primary antibodies were diluted in blocking solution and cells and antibodies were incubated O/N at 4 °C: rabbit anti-beta tubulin III (1:1000; Biolegend; 802,001), chicken anti-MAP2 (1:2500; Abcam; ab92434), mouse anti-TAU (1:500, Thermo Scientific; clone HT7 MN1000), mouse anti-APP-C-terminus (C1/6.1; 1:2000; Biolegend), mouse anti-Tau 3-repeat isoform RD3 (1:1000; Sigma-Aldrich; 8E6/C11), mouse anti-Tau 4-repeat isoform RD4 (1:500; Sigma-Aldrich; 1E1/A6). Thereafter, cells were washed with PBT and blocked for 10 min. Secondary antibodies conjugated to fluorophores − 647 or − 488 (Invitrogen; A-31571, Jackson Immunoresearch; 703-545-155, Invitrogen; A10042) were diluted in blocking buffer and applied for 1 h at RT, then washed and counterstained with 49,6-diamidino-2-phenylindole (DAPI; 1 μg/mL; Sigma) diluted in PBS (Life Technologies). Cells were washed three times with PBS (Life Technologies) and mounted using PVA-DABCO (Sigma-Aldrich).

### Immunofluorescence Staining on Tissue Sections

Formalin-fixated tissues were embedded in paraffin blocks and cut in 2–4-μm sections and mounted on superfrost plus slides. Sections were then deparaffinised in xylene O/N and rehydrated (30 min 99% EtOH; 20 min 96% EtOH; 10 min 70% EtOH; rinsed with distilled water). Antigens were retrieved by boiling in TEG buffer (10 mM Tris, 0.5 mM EGTA, pH 9) and aldehyde groups were shielded in 50 mM NH_4_Cl in PBS. Sections were rinsed 30 min in blocking solution 1% BSA, 0.2% gelatin in PBS. The primary antibodies were diluted in 0.1% BSA, 0.3% Triton X-100 in PBS and incubated O/N in a humidity chamber at 4 °C: mouse anti-Tau 3-repeat isoform RD3 (1:100; Sigma-Aldrich; 8E6/C11), mouse anti-Tau 4-repeat isoform RD4 (1:100; Sigma-Aldrich; 1E1/A6). The following day, the sections were rinsed three times and incubated for 30 min at room temperature with secondary antibodies conjugated to fluorophores − 647 or − 488 (Jackson Immunoresearch) followed by three washes in PBS and mounting of coverslips (VWR) using PVA-DABCO (Sigma-Aldrich).

### Microscopy

Quantification of the percentage of TUJ1 and MAP2-positive-induced neurons were obtained using Cellomics Array Scan (Array Scan VTI, Thermo Fischer) by applying the program ‘Target Activation’ and ‘Neuronal Profiling’. Objects were selected based on the intensity and size parameters set by the user. Images were obtained with a Zeiss LSM confocal lsm780 or Leica fluorescent microscope (Wetzlar, Germany).

### Protein Extraction and Western Blotting

Cells and tissues were lysed in RIPA lysis buffer (prepared in house; 50 mM Tris HCl, 150 mM NaCl 150 mM, Sodiumdeoxycholate 0.5%, 0.1% SDS, 1% NP40) containing protease inhibitors (c0mplete Mini, EDTA-free protease inhibitor cocktail; Roche) followed by sonication (Bioruptor®). Tissue samples were homogenised in RIPA buffer using a tissue-grinder before sonication. Homogenates were centrifuged at 5000*g* for 10 min at 4 °C. The supernatant was collected, and protein concentration was measured using Bradford protein assay (Bio-Rad) following the manufacturer’s protocol.

Proteins were separated by SDS-PAGE on 16.5% Tris/Tricine gels (Bio-Rad) for cell lysates, 7.5% Glycine gels (Bio-Rad) for conditioned media and 10% Glycine gels (Bio-Rad) for tissue lysates and were transferred to nitrocellulose or PVDF membranes, respectively (0.2 μm; Bio-Rad). The membranes were boiled in PBS (Life Technologies) for 5 min to unmask the epitopes and blocked in 5% skim milk (5 g Skim Milk (Difco™), 50 mL TBS-T containing 100 mL 10x Tris Buffered Saline (Fisher BioReagents), 900 mL water, 2 mL 100% Tween 20 (Sigma-Aldrich)) for 1 h prior to probing with primary antibody O/N: mouse anti-APP-C-terminus (C1/6.1; 1:2000; Biolegend), rabbit anti-APP-N-terminus (22C11; 1:2000; Calbiochem; MAB348), anti-beta-actin (1:10,000; Abcam; ab6276), mouse anti-Tau 3-repeat isoform RD3 (1:10,000; Sigma-Aldrich; 8E6/C11) and mouse anti-Tau 4-repeat isoform RD4 (1:2000; Sigma-Aldrich; 1E1/A6). Membranes were washed 4 × 30 min and applied with horse-radish peroxidase-conjugated secondary antibodies, Goat anti-mouse (1:2000; Dako) or Goat anti-rabbit (1:2000; Dako) for 1.5 h followed by additional 4 × 30 min of washing. Blots were developed using Clarity™ Enhanced Chemiluminescence (Bio-rad) by a film based (Konica Minolta) or digital imaging. For reprobing, membranes were stripped using Re-Blot Strong Solution (Calbiochem).

### ELISA

ELISA was performed on conditioned media using ELISA Aβ1-40 and 1-42 kits (Thermo Fisher Scientific) following the manufacturer’s protocol. The medium was harvested from three experiments at day 28 post-transduction, and 1 mM PMSF was added prior to storage at − 20 °C until analysis.

### RNA Extraction and cDNA Synthesis

RNA from fibroblasts and iNs were extracted using an automated RNA purification system, Maxwell® RSC simplyRNA Tissue Kit (Promega). Murine and porcine cerebral cortical tissues were homogenised in TriReagent (Sigma-Aldrich) by an RNAse-free tissue-grinder. RNA was extracted using chloroform and isopropanol precipitation. RNA concentration and purity were evaluated using NanoDrop 1000 version 3.7.1. (Thermo Fisher Scientific). Complementary DNA (cDNA) was synthesised from RNA using iScript™ cDNA Synthesis Kit (Bio-Rad) with a 1:1 mix of oligo(dT) and random hexamer primers.

### RT-PCR and RT-qPCR

RT-PCR was carried out using Q5 high-fidelity or OneTaq Hotstart polymerases (New England Biolabs) according to the manufacturer’s protocol. PCR products were run on a 2% agarose gel. RT-qPCR was performed on pre-diluted cDNA and mixed with LightCycler® 480 SYBR Green I Master (Roche). Relative gene expression was calculated by a standard curve method [39] using serial dilutions of a pool of cDNA from all samples present in the study. Expression levels were normalised to the geometric mean of *GAPDH* and *HPRT1* reference genes. Negative controls as cDNA synthesis without reverse transcriptase and water only samples were included. All runs were performed in triplicates. Primers used for RT-PCR and RT-qPCR are shown in Supplementary Table 4.

### RNA Sequencing and Bioinformatics

Raw sequencing reads from published RNA-sequencing (RNAseq) experiments were extracted as FASTQ from National Center for Biotechnology Information Short Read Archive using Galaxy (Version 2.8.1.3) [40].

For pig RNAseq data files (GSE146494), reads were trimmed using Trimmomatic [41] to remove the first 10 bases from the start of the read. Reads were aligned to the porcine genome build Sscrofa11.1 (Ensemble release 98) using HISAT2 aligner (v2.1.0) [42]. Transcript quantification and junction counts were generated from featureCounts (v1.6.3) [43] and the read counts were normalised for effective gene length, and sequencing depth to yield Transcripts Per Kilobase Million (TPM). The raw junction counts were normalised for sequencing depth. We extracted counts of *MAPT* (ENSSSCG00000017311) junctions exon 9–10 (X_9,10_, chr12, position 17,122,167 to 17,112,299), 9–11 (X_9,11_, chr12, position 17,122,167 to 17,108,761) and 10–11 (X_10,11_, chr12, position 17,112,207 to 17,108,761). The 3R splice form is represented by X_9,11_ and 4R by both X_9,10_ and X_10,11_. The percentage of *MAPT* 4R usage was subsequently calculated by the following: 100 ∗ (0.5 ∗ (X_9,10_ + X_10,11_))/(X_9,11_). The number of reads per exon in *MAPT* and *APP* genes was counted using DEXSeq-Count (v1.28.1.0), and differential exon usage was determined using DEXSeq (v1.28.1) tool [44]. Differentially expressed genes were determined from count tables using DEseq2 (v2.11.40.2) [45]. Disease and biofunction pathway analysis was performed using the QIAGEN Ingenuity Pathway Analysis software.

For human RNAseq data (E-MTAB-3037; GSE132154), reads were aligned to the human genome build hg19/GRCh38 (Ensemble release 94) using HISAT2 aligner (v2.1.0) [42]. Reads aligned to *MAPT* (ENSG00000186868, chr17, position 45,894,382 to 46,028,334) were visually inspected using IGV software. Junction counts were generated from featureCounts (v1.6.3) [43]. The raw junction counts were normalised for sequencing depth. We extracted counts of *MAPT* (ENSG00000186868) junctions exon 9–10 (X_9,10_, chr17, position 45,996,664 to 46,010,310), 9–11 (X_9,11_, chr17, position 45,996,664 to 46,014,243) and 10–11 (X_10,11_, chr17, position 46,010,402 to 46,014,243). The 3R splice form is represented by X_9,11_ and 4R by both X_9,10_ and X_10,11_. The percentage of *MAPT* 4R usage was subsequently calculated by the following: 100 ∗ (0.5 ∗ (X_9,10_ + X_10,11_))/(X_9,11_).

## Supplementary Information

ESM 1 (DOCX 179742 kb)

## Data Availability

The GEO accession number for RNAseq reported in this paper is GSE146494.
