# Supplementary material for: Directly Reprogrammed Neurons Express MAPT and APP Splice Variants Pertinent to Ageing and Neurodegeneration
Source: Mol Neurobiol. 2021 Jan 7;58(5):2075–87. doi: 10.1007/s12035-020-02258-w (PMC8018937; doi:10.1007/s12035-020-02258-w)
Supplement: Supplementary file 1 — (DOCX 179742 kb) [file 12035_2020_2258_MOESM1_ESM.docx]

# Supplementary Materials

Directly Reprogrammed Neurons Express *MAPT* and *APP* Splice Variants Pertinent to Ageing and Neurodegeneration

*Mette Habekost^1,2,*^, Per Qvist^1,3,4^, Mark Denham^1,2^, Ida E. Holm^5,6^ and Arne Lund Jørgensen^1^*

^1^ Department of Biomedicine, Aarhus University, 8000C Aarhus, Denmark

^2^ Danish Research Institute of Translational Neuroscience, Nordic EMBL Partnership for Molecular Medicine, Aarhus University, 8000C Aarhus, Denmark

^3^ iPSYCH, Lundbeck Foundation Initiative for Integrative Psychiatric Research, 8000C Aarhus, Denmark

^4^ Center for Genomics and Personalized Medicine, 8000C Aarhus, Denmark

^5^ Department of Pathology, Randers Hospital, 8930 Randers, Denmark

^6^ Department of Clinical Medicine, Aarhus University, 8000C Aarhus, Denmark

# 1 Supplementary Figures

## 1.1 Supplementary Figure 1

## 1.2 Supplementary Figure 2

## 1.3 Supplementary Figure 3

# 2 Supplementary Figure Legends

## 2.1 Supplementary Figure 1 Mapt Cell-Type Specific Isoform Expression during Mouse Cortical Development.

Immunohistochemical analysis of MAPT isoform expression in the cerebral cortex of the mouse at different ages (E14.5; P14; 8 weeks old) using 3R (RD3; 8E6/C11) and 4R (RD4; 1E1/A6) specific antibodies (green). Neurons identified using MAP2 specific antibody (red). E14.5 showed neuronal staining of 3R tau. The same was true for P14 but not 8 weeks. P14 and 8 weeks stained positive for 4R tau. The lower panel includes negative control staining using anti-mouse IgG (msIgG, green) and no primary antibody (anti-ch647, red) from equivalent mouse (blue) and pig (green) brain sections and ages (related to Figure 2). Scale bars: 50μm. E, embryonic day; P, postnatal day.

## 2.2 Supplementary Figure 2 Human Tau Isoform Expression in Adult Cortex and Induced Neurons

(A) RT-PCR of *APP* and *MAPT* isoform expression in adult human cortex tissue using species-specific RT-PCR primers. (B, C) Visual inspection using IGV software of alternative splicing of human *MAPT* in induced neurons converted from human fibroblasts using N2AA protocol (Foreskin fibroblast, age: 0years; n=3; red)(Mertens et al., 2015). (D, E) As in (B, C) but REST_sh-PBmPA protocol (Dermal fibroblast; age: adult; n=3; blue)(Birtele et al., 2019). Chromosomal location (chr17) and several reads supporting alternative splicing events are indicated. E; Exon.

## 2.3 Supplementary Figure 3 Tau Isoform Expression in Porcine Induced Neurons

Immunostaining of wild-type porcine iNs for 3R (green; upper panel) or 4R (green; lower panel) tau and MAP2 (red) after 28 days of conversion. Scale bars: 50μm.

# 3 Supplementary Tables

## 3.1 Supplementary Table 1 Human *MAPT* and *APP* exon-exon junction positions

| Chromosome | Gene/Exon id | Position | | Strand |
| --- | --- | --- | --- | --- |
| 17 | ENSG00000186868 | 45894382 | 46028334 | + |
| 17 | E00 | 45894382 | 45894686 | + |
| 17 | E01 | 45962321 | 45962470 | + |
| 17 | E02 | 45971859 | 45971945 | + |
| 17 | E03 | 45974385 | 45974471 | + |
| 17 | E04 | 45978375 | 45978440 | + |
| 17 | E05 | 45987040 | 45987095 | + |
| 17 | E07 | 45991460 | 45991586 | + |
| 17 | E09 | 45996399 | 45996664 | + |
| 17 | E10 | 46010310 | 46010402 | + |
| 17 | E11 | 46014243 | 46014324 | + |
| 17 | E12 | 46018618 | 46018730 | + |
| 17 | E13 | 46023956 | 46028334 | + |
| 21 | ENSG00000142192 | 25880550 | 26171128 | - |
| 21 | E18 | 25880550 | 25881771 | - |
| 21 | E17 | 25891722 | 25891868 | - |
| 21 | E16 | 25897573 | 25897673 | - |
| 21 | E15 | 25905024 | 25905077 | - |
| 21 | E14 | 25911741 | 25911962 | - |
| 21 | E13 | 25954590 | 25954689 | - |
| 21 | E12 | 25955627 | 25955755 | - |
| 21 | E11 | 25975070 | 25975228 | - |
| 21 | E10 | 25975954 | 25976028 | - |
| 21 | E09 | 25982344 | 25982477 | - |
| 21 | E08 | 25997360 | 25997416 | - |
| 21 | E07 | 26000015 | 26000182 | - |
| 21 | E06 | 26021840 | 26022042 | - |
| 21 | E05 | 26051000 | 26051193 | - |
| 21 | E04 | 26053236 | 26053348 | - |
| 21 | E03 | 26089943 | 26090072 | - |
| 21 | E02 | 26111979 | 26112146 | - |
| 21 | E01 | 26170564 | 26170770 | - |

E; Exon, +; Forward strand, -; Reverse strand. GRCh38.

## 3.2 Supplementary Table 2 Porcine *MAPT* and *APP* exon-exon junction positions

| Chromosome | Gene/Exon id | Position | | Strand |
| --- | --- | --- | --- | --- |
| 12 | ENSSSCG00000017311 | 17095587 | 17211368 | - |
| 12 | E13 | 17098267 | 17102284 | - |
| 12 | E12 | 17107022 | 17107134 | - |
| 12 | E11 | 17108680 | 17108761 | - |
| 12 | E10 | 17112207 | 17112299 | - |
| 12 | E09 | 17122167 | 17122432 | - |
| 12 | E07 | 17126168 | 17126300 | - |
| 12 | E05 | 17131001 | 17131056 | - |
| 12 | E04 | 17140057 | 17140122 | - |
| 12 | E03 | 17143403 | 17143510 | - |
| 12 | E02 | 17146073 | 17146159 | - |
| 12 | E01 | 17150624 | 17150743 | - |
| 12 | E00 | 17211241 | 17211367 | - |
| 13 | ENSSSCG00000012022 | 189434869 | 189716057 | - |
| 13 | E18 | 189435283 | 189436061 | - |
| 13 | E17 | 189446780 | 189446926 | - |
| 13 | E16 | 189450338 | 189450438 | - |
| 13 | E15 | 189457551 | 189457604 | - |
| 13 | E14 | 189466429 | 189466650 | - |
| 13 | E13 | 189505179 | 189505278 | - |
| 13 | E12 | 189506106 | 189506234 | - |
| 13 | E11 | 189523366 | 189523524 | - |
| 13 | E10 | 189524221 | 189524295 | - |
| 13 | E09 | 189528351 | 189528484 | - |
| 13 | E08 | 189541274 | 189541330 | - |
| 13 | E07 | 189543972 | 189544139 | - |
| 13 | E06 | 189561008 | 189561210 | - |
| 13 | E05 | 189597995 | 189598188 | - |
| 13 | E04 | 189600044 | 189600156 | - |
| 13 | E03 | 189631880 | 189632009 | - |
| 13 | E02 | 189654367 | 189654534 | - |
| 13 | E01 | 189715855 | 189716057 | - |

E; Exon, +; Forward strand, -; Reverse strand. Sscrofa11.1.

## 3.3 Supplementary Table 3 Murine *Mapt* and *App* exon-exon junction positions

| Chromosome | Gene/Exon id | Position | | Strand |
| --- | --- | --- | --- | --- |
| 11 | ENSMUSG00000018411 | 104231390 | 104328219 | + |
| 11 | E00 | 104231390 | 104231607 | + |
| 11 | E01 | 104282368 | 104282480 | + |
| 11 | E02 | 104287123 | 104287209 | + |
| 11 | E03 | 104289908 | 104289994 | + |
| 11 | E04 | 104294859 | 104294924 | + |
| 11 | E05 | 104302361 | 104302410 | + |
| 11 | E07 | 104306626 | 104306758 | + |
| 11 | E09 | 104310269 | 104310534 | + |
| 11 | E10 | 104318157 | 104318249 | + |
| 11 | E11 | 104321351 | 104321432 | + |
| 11 | E12 | 104322428 | 104322540 | + |
| 11 | E13 | 104327972 | 104328219 | + |
| 16 | ENSMUSG00000022892 | 84949685 | 85173766 | - |
| 16 | E18 | 84949685 | 84955432 | - |
| 16 | E17 | 84962722 | 84962868 | - |
| 16 | E16 | 84965670 | 84965770 | - |
| 16 | E15 | 84971408 | 84971461 | - |
| 16 | E14 | 84978064 | 84978285 | - |
| 16 | E13 | 85013627 | 85013726 | - |
| 16 | E12 | 85015232 | 85015360 | - |
| 16 | E11 | 85024853 | 85025011 | - |
| 16 | E10 | 85025423 | 85025497 | - |
| 16 | E09 | 85030232 | 85030365 | - |
| 16 | E08 | 85040251 | 85040307 | - |
| 16 | E07 | 85043576 | 85043743 | - |
| 16 | E06 | 85056324 | 85056526 | - |
| 16 | E05 | 85079840 | 85080033 | - |
| 16 | E04 | 85082776 | 85082888 | - |
| 16 | E03 | 85103206 | 85103335 | - |
| 16 | E02 | 85120530 | 85120697 | - |
| 16 | E01 | 85173502 | 85173704 | - |

E; Exon, +; Forward strand, -; Reverse strand. GRCm38.

## 3.4 Supplementary Table 4 Primers used in this study

| ID | Primer sequence (5’-3’) | Species | Annealing temp. (^o^C) | Amplicon (bp) | Application |
| --- | --- | --- | --- | --- | --- |
| N-Tau | E1 F: TCC GCT GTC CTC TTC TGT C | mm | 52  OneTaq | N0: 253  N1: 341  N2: 428 | 1 |
|  | E5 R: TTC TCG TCA TTT CCT GTC C |  |  |  |  |
| R-Tau | E7 F: CCC TAA GAC TCC TCC AGG GT | mm | 56  OneTaq | 3R: 328  4R: 421 | 1 |
|  | E11 R: CAC TTT GCT CAG GTC CAC CGG C |  |  |  |  |
| N-Tau | E1 F: GAT CAT GCT CAG GGG GAC TA | ss | 62  Q5 | N0: 179  N1: 266  N2: 374 | 1 |
|  | E5 R: TTG TCA TCG GGT CCA GTG CC |  |  |  |  |
| R-Tau | E9 F: CCA AGA AAG TGG CAG TGG TC | ss | 62  Q5 | 3R: 285  4R: 192 | 1 |
|  | E11 R: CCT TGC TCA GAT CCA CTG GT |  |  |  |  |
| N-Tau | E1 F: TGA ACC AGG ATG GCT GAG C | hs | 62  Q5 | N0: 254  N1: 341  N2: 428 | 1 |
|  | E5 R: TTG TCA TCG CTT CCA GTC C |  |  |  |  |
| R-Tau | E9 F: AAG ATC GGC TCC ACT GAG AA | hs | 62  Q5  +GC enhancer | 3R: 316  4R: 223 | 1 |
|  | E12 F: GGA CGT GGG TGA TAT TGT CC |  |  |  |  |
| App | E6 F: GGC TGA TGT TGA GGA AGA GG | mm | 54  OneTaq | 770: 637  751: 580  695: 412 | 1 |
|  | E10 R: TGG ATA ACG GCC TTC TTG TC |  |  |  |  |
| APP | E6 F: AGG AAC CCT ATG AAG AGG CC | ss | 62  Q5 | 770: 534  751: 477  695: 309 | 1 |
|  | E10/11 R: CCA CTT TCT CCT GGA AAT GC |  |  |  |  |
| APP | E6 F: CAC AGA GAG AAC CAC CAG CA | hs | 62  Q5  +GC enhancer | 770: 364  751: 532  695: 589 | 1 |
|  | E11 R: CAC TCT GGC CAT GTG TGT CT |  |  |  |  |
| 3R-Tau | E9/11: CGG GAA GGT GCA AAT AGT CT | ss | 60 | 169 | 2 |
|  | E12 R: GTT ATC CAG GGA CCC GAT CT |  |  |  |  |
| 4R-Tau | E10/11 F: GCG GCA GTG TGC AAA TAG T | ss | 60 | 184 | 2 |
|  | E12 R: GGG ACG TGG GTG ATG TTA TC |  |  |  |  |
| HPRT1 | F: GGA CTT GAA TCA TGT TTG TG | ss | 60 | 91 | 2 |
|  | R: CAG ATG TTT CCA AAC TCA AC |  |  |  |  |
| GAPDH | F: ACA CTC ACT CTT CTA CCT TTG | ss | 60 | 90 | 2 |
|  | R: CAA ATT CAT TGT CGT ACC AG |  |  |  |  |
|  | R: TGG GGA GGC TAT AGA AAA TAA GG |  |  |  |  |

E; Exon, F; Forward primer, R; Reverse Primer. Species: hs; homo sapiens, mm; mus musculus, ss; sus scrofa. Application: 1; RT-PCR, 2; RT-qPCR.

# 4 References

Birtele, M., Sharma, Y., Kidnapillai, S., Lau, S., Stoker, T.B., Barker, A., Ottosson, D.R., Drouin-ouellet, J., Parmar, M., 2019. Dual modulation of neuron-specific microRNAs and the REST complex promotes functional maturation of human adult induced neurons. FEBS Lett. 593, 3370–3380. https://doi.org/10.1002/1873-3468.13612

Mertens, J., Paquola, A.C.M., Ku, M., Hatch, E., Böhnke, L., Ladjevardi, S., McGrath, S., Campbell, B., Lee, H., Herdy, J.R., Gonçalves, J.T., Toda, T., Kim, Y., Winkler, J., Yao, J., Hetzer, M.W., Gage, F.H., 2015. Directly Reprogrammed Human Neurons Retain Aging-Associated Transcriptomic Signatures and Reveal Age-Related Nucleocytoplasmic Defects. Cell Stem Cell 17, 705–718. https://doi.org/10.1016/j.stem.2015.09.001
